# Screen for *IDH1*, *IDH2*, *IDH3*, *D2HGDH* and *L2HGDH* Mutations in Glioblastoma

**DOI:** 10.1371/journal.pone.0019868

**Published:** 2011-05-23

**Authors:** Daniel Krell, Mawuelikem Assoku, Malcolm Galloway, Paul Mulholland, Ian Tomlinson, Chiara Bardella

**Affiliations:** 1 Molecular and Population Genetics Laboratory, Wellcome Trust Centre for Human Genetics, University of Oxford, Oxford, United Kingdom; 2 Department of Medical Oncology, University College London Hospitals, London, United Kingdom; 3 Department of Cellular Pathology, The Royal Free Hospital, London, United Kingdom; University of Pittsburgh School of Medicine, United States of America

## Abstract

Isocitrate dehydrogenases (IDHs) catalyse oxidative decarboxylation of isocitrate to α-ketoglutarate (α-KG). IDH1 functions in the cytosol and peroxisomes, whereas IDH2 and IDH3 are both localized in the mitochondria. Heterozygous somatic mutations in *IDH1* occur at codon 132 in 70% of grade II–III gliomas and secondary glioblastomas (GBMs), and in 5% of primary GBMs. Mutations in *IDH2* at codon 172 are present in grade II–III gliomas at a low frequency. *IDH1* and *IDH2* mutations cause both loss of normal enzyme function and gain-of-function, causing reduction of α-KG to D-2-hydroxyglutarate (D-2HG) which accumulates. Excess hydroxyglutarate (2HG) can also be caused by germline mutations in D- and L-2-hydroxyglutarate dehydrogenases (*D2HGDH* and *L2HGDH*). If loss of IDH function is critical for tumourigenesis, we might expect some tumours to acquire somatic *IDH3* mutations. Alternatively, if 2HG accumulation is critical, some tumours might acquire somatic *D2HGDH* or *L2HGDH* mutations. We therefore screened 47 glioblastoma samples looking for changes in these genes. Although *IDH1* R132H was identified in 12% of samples, no mutations were identified in any of the other genes. This suggests that mutations in *IDH3*, *D2HGDH* and *L2HGDH* do not occur at an appreciable frequency in GBM. One explanation is simply that mono-allelic *IDH1* and *IDH2* mutations occur more frequently by chance than the bi-allelic mutations expected at *IDH3*, *D2HGDH* and *L2HGDH*. Alternatively, both loss of IDH function and 2HG accumulation might be required for tumourigenesis, and only *IDH1* and *IDH2* mutations have these dual effects.

## Introduction

Gliomas are the most common primary brain tumour, accounting for 70% of all primary central nervous system neoplasms. They show wide diversity with respect to location, morphology, genetic status and response to therapy.

Grade I gliomas occur more in children than in adults, are generally curable with complete surgical resection and rarely evolve into higher-grade lesions. WHO grade II or III gliomas are invasive, progress to higher-grade lesions and have a poor outcome. Glioblastoma (GBM), the most common and most malignant glioma, has a very poor prognosis and may develop rapidly without evidence of a less malignant precursor lesion (primary glioblastoma), or less commonly through progression from a lower grade tumour (secondary glioblastoma) [1], [2].

Isocitrate dehydrogenases (IDHs) catalyse the oxidative decarboxylation of isocitrate to α-ketoglutarate (α-KG) and reduce NAD^+^ or NADP^+^ to NADH or NADPH. IDH1 and IDH2 are homodimeric, NADP^+^-dependent enzymes that share considerable sequence similarity and an almost identical protein structure [3]. IDH3 is a heterotetramer composed of two α, one β and one γ subunit. IDH3 is a NAD^+^-dependent enzyme. IDH1 is localised to the cytoplasm and peroxisomes, it is highly expressed in the liver and to a lesser level in other tissues [4]. As well as being involved in catalysing the conversion of isocitrate to α-KG, IDH1 is thought to play a role in cellular metabolic processes such as lipid and glucose metabolism [5], [6], and has been shown to be involved in cellular defence against reactive oxygen species and radiation [7], [8], [9].

IDH2 is localised to the mitochondria, is highly expressed in heart, muscle, activated lymphocytes and moderately in other tissues [4]. IDH2 plays a key role in the regulation of the tricarboxylic acid cycle (TCA) and like IDH1, has been shown to have a protective role against insults such as oxidative stress [9], [10], [11].

IDH3 is also localised to the mitochondria and plays a central role in the TCA cycle.

In 2008, a genome-wide sequencing study identified somatic mutations in *IDH1* in 18 (12%) of 149 patients with GBM [12] and it was subsequently found that GBMs without *IDH1* mutations often have mutations affecting *IDH2* [13]. Both *IDH1* and *IDH2* mutations are more frequent in grade II-III gliomas and secondary glioblastoma (70–75%) than in primary glioblastoma (5%) [14], are present at higher frequencies in younger patients, and are associated with a relatively favourable prognosis [13], [15]. *IDH1* and *IDH2* mutations are mono-allelic, somatic, missense changes. Mutations in *IDH1* almost always affect R132, which is the binding site for isocitrate [12]. Mutations in *IDH2* exclusively affect R172 and R140; the former of these arginines is analogous to *IDH1* R132 [13] [16].

Initial investigations demonstrated that mutated IDH1 had a reduced affinity for isocitrate [17]. Furthermore, expression of mutant *IDH1* in cultured cells has been shown to reduce the formation of the enzyme products, α-KG and NADPH [13], [17], [18]. These structural and biochemical findings suggested that *IDH* mutation results in loss of function, the most plausible mechanism being a dominant negative effect through the formation of catalytically inactive heterodimers of mutant and wild type proteins [17]. Deficiency of α-KG might lead to defective function of α-KG-dependent enzymes such as HIF prolyl hydroxylases.

Subsequent evidence has emerged suggesting that mutant IDH1 and IDH2 are not simply inactive enzymes, but instead possess novel enzymatic activity [16], [19], [20]. Dang *et al*. demonstrated that mutant, but not wild type, IDH1 catalyses the reduction of α-KG to D-2HG while converting NADPH to NADP^+^. D-2HG accumulates in glioblastoma cells with *IDH1* R132H mutations [19]. The role of 2HG in tumourigenesis is unclear, although it may act as an oncometabolite, perhaps competitively inhibiting α-KG-dependent enzymes [21] [22]. One of the rationales for a role of 2HG in tumour formation is derived from the observation of patients suffering from hereditary 2-hydroxyglutaric aciduria. This is a rare inherited metabolic disorder, caused by homozygous inactivating germline 2-hydroxyglutarate dehydrogenase (*2HGDH*) mutations, that is weakly associated with brain tumours.

If IDH loss-of-function alone is critical for tumourigenesis, we might expect *IDH3* mutations to occur in brain tumours, including those sub-types with relatively low frequencies of *IDH1* and *IDH2* mutations. Alternatively, if accumulation of 2HG alone is critical, we might expect mutations in *D2HGDH* and/or *L2HGDH*. A further, more speculative line of reasoning is that, if patients with mutations in *IDH1* and *IDH2* have normal D2HGDH and L2HGDH function, excess 2HG should simply be converted back to α-KG by 2HGDH. The failure of this to occur might be due to saturation of the 2HGDH enzymes, but raises the possibility that patients with *IDH1* and *IDH2* mutations might also require inactivating mutations in *D2HGDH* and *L2HGDH* for 2HG to accumulate.

We therefore analysed a set of GBM for mutations in *IDH1*, *IDH2*, *IDH3*, *D2HGDH* and *L2HGDH*.

## Results and Discussion

**Figure 1:**
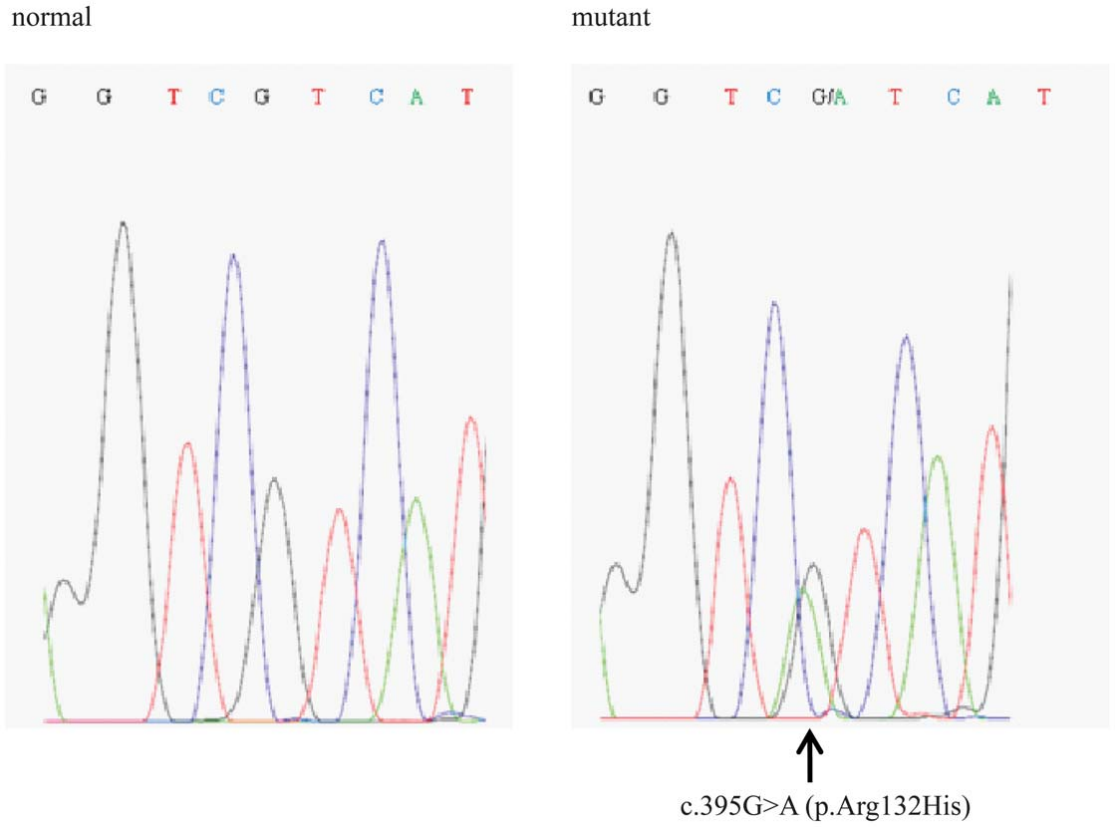
Identification of the G395A mutation at codon 132 by sequence analysis of the *IDH1* gene. The electropherogram shows a representative example of the heterozygous, single base G-to-A substitution at nucleotide position 395 of the *IDH1* gene (right panel) and the corresponding wild-type sequence (left panel). The mutation was detected in 6 out of 47 glioblastomas analyzed.

We studied 47 glioblastomas (WHO grade IV). Heterozygous mutations of *IDH1* were found in 6/47 tumours (12%). All 6 mutations were single base substitutions c.395G>A occurring at residue R132, resulting in an arginine to histidine (p.R132H) substitution (Figure 1). This frequency is consistent with previously described data. No mutations were found in *IDH2*, in keeping with the lower frequency of such changes than mutations in *IDH1*. We sequenced all the exons of *IDH3A*, *IDH3B* and *IDH3G*, which encode the α, β, and γ subunits of the IDH3 heterotetramer, and all the exons of *D2HGDH* and *L2HGDH*. In all cases examined we did not find any mutations. Known SNPs were found within each gene (details not shown).

**Figure 2:**
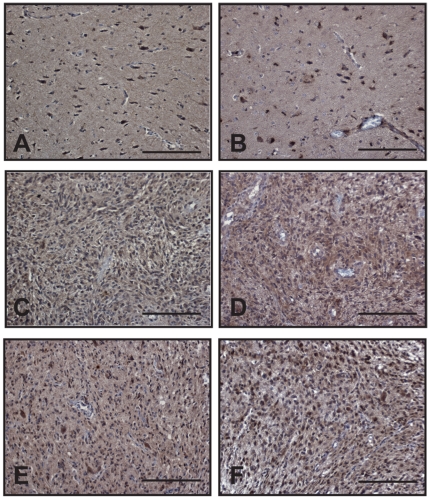
D2HGDH and L2HGDH are expressed in wildtype *IDH1/2* and *IDH1* R132H glioblastomas. Expression of D2HGDH and L2HGDH in normal brain is shown respectively in panels A and B. D2HGDH and L2HGDH were detected in brain tumour samples carrying either wild-type *IDH1/IDH2* (C and D) or mutated *IDH1* allele (E and F) by immunohistochemistry. Scale bar represents 50 µm in all panels.

We then wondered whether *D2HGDH* or *L2HGDH* might be inactivated by other mechanisms such as promoter methylation or miRNA over-expression. We therefore evaluated the expression of the corresponding proteins in human glioblastomas samples (Figure 2). D2HGDH and L2HGDH were detected in GBMs carrying wild type *IDH1* and *IDH2* genes, as well as in *IDH1*-mutants tumours. It is highly unlikely that the loss of D2HGDH or L2HGDH expression plays an important role in the pathogenesis of human GBMs.

Mutations in *IDH3, D2HGDH* and *L2HGDH* (or loss of expression of the 2HGDHs) do not therefore occur at an appreciable frequency in GBM. One explanation is simply that mono-allelic *IDH1* and *IDH2* mutations occur more frequently by chance than the bi-allelic mutations expected at *IDH3*, *D2HGDH* and *L2HGDH*. Alternatively, both loss of IDH function and 2HG accumulation are required for tumourigenesis, and only *IDH1* and *IDH2* mutations have these dual effects. It is also possible that 2HG accumulation is an epiphenomenon and that IDH3 loss-of-function does not lead to sufficient deficiency of α-KG to promote tumourigenesis. There might also be an unknown mechanism of tumourigenesis that is specific to defects in IDH1 or IDH2.

Moreover our data suggest that in patients with mutations of *IDH1* and *IDH2* and normal D2HGDH and L2HGDH function, the excess 2HG is not converted back to α-KG by 2HGDH, perhaps due to the saturation of the latter enzyme. A recent report identified heterozygous germline *IDH2* mutations in patients with idiopathic D-2-hydroxyglutaric aciduria, carrying no mutations in *D2HGDH* and consistently increased D-2HG levels in body fluids [23]; interestingly, the patient did not develop brain tumours.

The mechanisms by which *IDH1* and *IDH2* mutations cause tumourigenesis remain largely unclear, as do the putative alternative functional deficiencies in *IDH*-wildtype gliomas. However, even if 2HG has no direct pathogenic role it has potential as a specific marker of *IDH1* or *IDH2* mutations, to diagnose or monitor *IDH*-mutant glioma [24].

## Materials and Methods

### Sample collection

All brain tumours were obtained from the neuropathology department at The Royal Free Hospital, Hampstead, London. All 47 samples analysed were confirmed to be WHO grade IV glioblastoma. We have worked solely on anonymously samples. Study of these has been approved by Oxfordshire REC B 05/Q1605/66.

### DNA extraction

DNA was extracted from paraffin embedded samples using DNeasy Blood and Tissue from Quiagen^R^ (Alameda, CA), following the manufacturer's instructions.

### Sequencing analysis

Mutation screening of each gene was performed by direct sequencing of genomic DNA in forward and reverse orientations using the Applied Biosystems BigDye terminator reaction kit and the AB 3730xl sequencing machine (Applied Biosystems, Foster City, CA). Primer sequences were designed to encompass the coding region and splice sites of exon 4 of *IDH1* and *IDH2*, and all exons of *IDH3A* (RefSeq: NM_005530.2), *IDH3B* (RefSeq: NM_174855.1), *IDH3G* (RefSeq: NM_004135.2 for the transcript variant 1 and RefSeq: NM_174869.1 for the transcript variant 2), *L2HGH* (RefSeq: NM_024884.2) and *D2HGDH* (RefSeq: NM_024884.2). Primer sequences and PCR conditions are available on request.

### Immunohistochemistry

Formalin-fixed, paraffin-embedded tissue sections (4 µm) were de-waxed in xylene and rehydrated through graded alcohols to water. Endogenous peroxidase was blocked using 1.6% H_2_O_2_ for 20 minutes. For antigen retrieval, sections were pressure cooked in 10 mmol/L citrate buffer (pH6.0) for 5 minutes. Sections were blocked with 10% serum for 30 minutes. Slides were incubated with primary polyclonal antibodies anti human D2HGDH (ProteinTech Group, Chicago, IL 60612, 1∶50), or anti human L2HGDH (ProteinTech Group, Chicago, IL 60612, 1∶100) for 1 hour. Goat anti-rabbit secondary antibody was applied for one hour at room temperature. Sections were then incubated in ABC (Vector labs) for 30 minutes. DAB solution was applied for 2–5 minutes and development of the colour reaction was monitored microscopically. Slides were counterstained with haematoxylin, dehydrated, cleared and then mounted. Images were taken at 20× magnification.
